# Nigeria’s public health response to the COVID-19 pandemic: January to May 2020

**DOI:** 10.7189/jogh.10.020399

**Published:** 2020-12

**Authors:** Chioma Dan-Nwafor, Chinwe Lucia Ochu, Kelly Elimian, John Oladejo, Elsie Ilori, Chukwuma Umeokonkwo, Laura Steinhardt, Ehimario Igumbor, John Wagai, Tochi Okwor, Olaolu Aderinola, Nwando Mba, Assad Hassan, Mahmood Dalhat, Kola Jinadu, Sikiru Badaru, Chinedu Arinze, Abubakar Jafiya, Yahya Disu, Fatima Saleh, Anwar Abubakar, Celestina Obiekea, Adesola Yinka-Ogunleye, Dhamari Naidoo, Geoffrey Namara, Saleh Muhammad, Oladipupo Ipadeola, Chinenye Ofoegbunam, Oladipo Ogunbode, Charles Akatobi, Matthias Alagi, Rimamdeyati Yashe, Emily Crawford, Oyeladun Okunromade, Everistus Aniaku, Sandra Mba, Emmanuel Agogo, Michael Olugbile, Chibuzo Eneh, Anthony Ahumibe, William Nwachukwu, Priscilla Ibekwe, Ope-Oluwa Adejoro, Winifred Ukponu, Adebola Olayinka, Ifeanyi Okudo, Olusola Aruna, Fatima Yusuf, Morenike Alex-Okoh, Temidayo Fawole, Akeem Alaka, Hassan Muntari, Sebastian Yennan, Rhoda Atteh, Muhammad Balogun, Ndadilnasiya Waziri, Abiodun Ogunniyi, Blessing Ebhodaghe, Virgile Lokossou, Mohammed Abudulaziz, Bimpe Adebiyi, Akin Abayomi, Ismail Abudus-Salam, Sunday Omilabu, Lukman Lawal, Mohammed Kawu, Basheer Muhammad, Aminu Tsanyawa, Festus Soyinka, Tomi Coker, Olaniran Alabi, Tony Joannis, Ibrahim Dalhatu, Mahesh Swaminathan, Babatunde Salako, Ibrahim Abubakar, Braka Fiona, Patrick Nguku, Sani H Aliyu, Chikwe Ihekweazu

**Affiliations:** 1 Nigeria Centre for Disease Control, Abuja, Nigeria; 2 African Field Epidemiology Network, Abuja, Nigeria; 3 Department of Community Medicine, Alex Ekwueme Federal University Teaching Hospital Abakaliki, Ebonyi State, Nigeria; 4 Center for Global Health, Centers for Disease Control and Prevention, FCT Abuja, Nigeria; 5 School of Public Health, University of the Western Cape, Cape Town, South Africa; 6 World Health Organisation, Abuja, Nigeria; 7 Resolve to Save Lives Resolve to Save Lives (Vital Strategies), Abuja, Nigeria; 8 World Bank, Nigeria Country Office, Abuja, Nigeria; 9 Tony Blair Institute, Tony Blair Institute for Global Change, London, UK; 10 George Town University Center for Global Health Practice and Impact, Abuja, Nigeria; 11 Public Health England International Health Regulations (IHR) Strengthening Project, British High Commission, Abuja, Nigeria; 12 Nigeria Port Health Services, Federal Ministry of Health Abuja, Nigeria; 13 ECOWAS Regional Center for Disease Surveillance and Control, Abuja-Nigeria; 14 Africa Centers for Disease Control and Prevention, African Union Commission, Addis Ababa Ethiopia; 15 Department of Hospital Services, Federal Ministry of Health Abuja, Federal Secretariat Abuja, Nigeria; 16 Lagos State Ministry of Health Ikeja, Lagos, Nigeria; 17 College of Medicine, University of Lagos Teaching Hospital Lagos, Nigeria; 18 Health and Human Services Secretariat Federal, Capital Territory Administration, Abuja, Nigeria; 19 Kano State Ministry of Health, Kano, Nigeria; 20 Ogun State Ministry of Health Abeokuta, Nigeria; 21 Federal Ministry of Agriculture and Rural Development, Federal Secretariat Abuja, Nigeria; 22 National Veterinary Research Institute Vom, Plateau State, Nigeria; 23 Nigerian Institute of Medical Research, Lagos, Nigeria; 24 Institute of Global Health, University College London, London, UK; 25 Department of Infectious Diseases, Cambridge University Hospitals, Cambridge, UK

The novel coronavirus disease 2019, COVID-19, which is caused by severe acute respiratory syndrome virus 2 (SARS-CoV-2) [1] was first reported in December 2019 by Chinese Health Authorities following an outbreak of pneumonia of unknown origin in Wuhan, Hubei Province [2,3]. SARS-CoV-2 is likely of zoonotic origin, similar to SARS and Middle East Respiratory Syndrome (MERS), and transmitted between humans through respiratory droplets and fomites. Since its emergence, it has rapidly spread globally [4].

The World Health Organisation (WHO) declared the novel coronavirus outbreak a Public Health Emergency of International Concern (PHEIC) on January 30, 2020 [5]. As COVID-19 spread to more countries and caused an increasing number of deaths, WHO led a mission to China with a team of experts from eight countries including Nigeria, to determine the extent of the outbreak, robustness of the response and identify best practices. Subsequently, on March 11, 2020, WHO declared COVID-19 a pandemic, calling for countries to take urgent and aggressive action [6,7]. There is a growing body of literature on innovative country-level responses to COVID-19, which describes the current approaches to developing policies and strategies relevant to the pandemic [8,9]. As the most populous country in Africa, and the sixth most populous in the world, Nigeria plays a significant role in the global response. Nigeria’s epidemic response is carried out in the context of a fragile and under-resourced existing health delivery system, and complicated by economic, political, social, and security issues throughout the country. Yet, confronting epidemics is not new to Nigeria. The 2014 Ebola epidemic sensitized the health system, government, and communities to the menacing impact of highly infectious diseases such as COVID-19 and the need to mount rapid, proactive measures [10]. In addition, the emergence of a strengthened Nigeria Centre for Disease Control (NCDC) has enhanced diagnostic and surveillance capacity in the country. We report the emergence of COVID-19 in Nigeria, describing the public health response up till May 2020.

**Figure:**
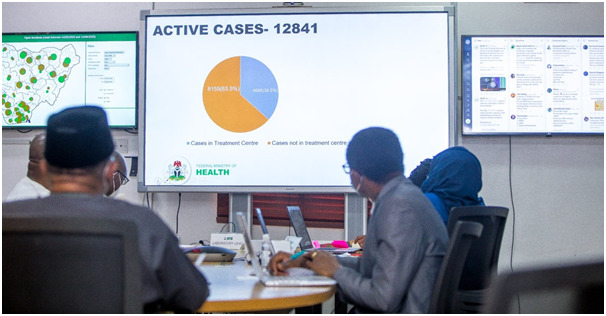
Photo: Regular data monitoring at Nigeria COVID-19 EOC to guide public health response (Source: Nigeria Centre for Disease Control, used with permission).

## COVID-19 IN NIGERIA

On February 27, 2020, the Federal Ministry of Health confirmed the first COVID-19 case in Ogun State, Nigeria, making the country the third country in Africa to recognise an imported COVID-19 case after Egypt and Algeria. The index case occurred in an Italian citizen who flew from Milan, Italy to Lagos, Nigeria on February 24, 2020, and travelled on to his company site in Ogun State the same day in a private vehicle. On February 26, 2020, he presented at the company clinic with symptoms consistent with COVID-19 and was referred to the Infectious Disease Hospital (IDH) in Lagos where a COVID-19 diagnosis was confirmed by real-time reverse transcription polymerase chain reaction (RT-PCR) on February 27, 2020.

A total of 216 contacts in Lagos and Ogun States, including the passengers on the February 24 air flight, were identified for 14-day follow-up, with 40 of these contacts identified as high-risk. Eleven days later, an asymptomatic contact of the index case in Ogun State was confirmed as the nation’s second case of COVID-19.

**Figure 1:**
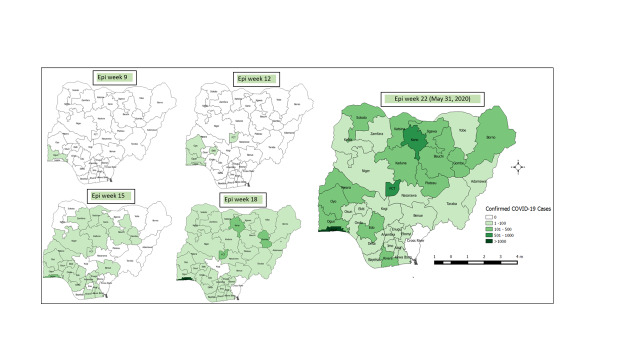
Trend of States reporting COVID-19 confirmed cases in Nigeria from Epidemiological Weeks 9, 12, 15, 18 to 22 (May 31, 2020).

The epidemiology of COVID-19 in Nigeria has since evolved, with cases identified in 35 of 36 states in the country, plus the Federal Capital Territory (FCT) (**Figure 1**). Although Lagos State was initially the epicentre of the outbreak, Kano State and the FCT have now joined Lagos State as high-burden states, contributing 64 · 5% of the cumulative total cases in Nigeria by the end of May 2020. Between February 27 and May 31, 2020, 63 882 persons have been tested for COVID-19 in Nigeria, of which 10 162 (15.9%) were confirmed as being infected with SARS-CoV-2 by RT-PCR. Males appear to be disproportionately affected accounting for 67.7% (6,882) of the confirmed cases. A total of 287 deaths have been recorded among the confirmed COVID-19 cases, resulting in an observed case fatality ratio (CFR) of approximately 2.8% [11].

## NIGERIA’S PREPAREDNESS AND RESPONSE TO COVID-19

### Nigeria’s pre-outbreak preparedness measures

Following reports of the coronavirus disease in Wuhan, China in December 2019, the NCDC published a notification of a new virus on its website on January 7, 2020. Subsequently, on January 26, 2020, the NCDC established a multisectoral National Coronavirus Preparedness Group (NCPG) in order to ensure a cohesive and effective coordination of the country’s preparedness efforts (**Table 1**). The NCPG met daily to review global COVID-19 epidemiology, assess the risk of spread, and initiate measures to strengthen the country’s preparedness for early detection and timely response in the event of a COVID-19 outbreak in Nigeria. An inter-ministerial Multisectoral Technical Working Group was inaugurated at the Federal Ministry of Health on January 31, 2020, to further strengthen preparedness.

**Table 1:** COVID-19 preparedness and response public health actions/interventions in Nigeria, January – May 2020

Outbreak phase	Public actions/health interventions	Strategy
**Pre-outbreak (January 1 – February 27)**	• Inauguration of multisectoral National Coronavirus Preparedness Group (CPG) by NCDC	**Prevention**
	• Inauguration of inter-Ministerial Coordination Committee by Honourable Minister for Health	
	• Review of Nigeria’s Pandemic Influenza Preparedness and Response Plan	
	• Activation of interim Medical Countermeasure Plan	
	• Conduct of table-top Logistic Capacity Assessment for COVID-19	
	• Training and capacity building of health care workers on infection prevention and control (IPC), sample collection and testing and clinical management of COVID-19	
	• Designation of three molecular Laboratories for COVID-19 testing	
	• Designation of COVID-19 treatment centres	
	• Points of Entry (PoE) surveillance at international borders including airports and land crossings	
	• Conduct of COVID-19 simulation exercise	
**Outbreak (February 27 – May 31)**		
**Stemming initial cases (February 27 – March 17)**	• Inauguration of national multisectoral COVID-19 Emergency Operation Centre (EOC)	**Containment**
	• Development of national Incident Action Plan and State Pre-Incident Action Plan	
	• Development of guidelines for surveillance, IPC, case management, schools, mass gatherings etc.	
	• Pre-positioning of COVID-19 response materials in 36 States and the Federal Capital Territory (FCT)	
	• Genetic sequencing of the index case conducted	
	• Establishment of Presidential Task Force (PTF) on COVID-19	
	• Deployment of Rapid Response Teams (RRTs) to support response activities in Lagos and Ogun	
	• Tracing of contact of confirmed cases	
	• Points of entry screening in high priority states with international airports including Lagos State	
	• Intensive risk communication including press releases, radio jingles, media appearances, social media	
	• Establishment of NCDC COVID-19 microsite	
**Addressing initial clusters of cases (March 18 – April 10)**	• Implementation of domestic and international travel restriction	**Suppression/Containment**
	• Strengthening and expansion of COVID-19 laboratory diagnostic capacity from five to 18	
	• Strengthening and expansion of COVID-19 treatment centres	
	• Domestic and international travel restrictions	
	• Lockdown of non-essential activities and stay-at-home orders in the FCT, Lagos and Ogun States	
	• Implementation of community active case search in Lagos and FCT	
**Focus on community transmission (April 11 – To Date)**	• Revision of the national case definition to increase case detection	**Mitigation**
	• Inter-State border screening in FCT, Lagos, and Ogun States	
	• Mid-action review meeting conducted	
	• Mandatory institutional quarantine and testing for international returnees	

Measures instituted by the NCPG included strengthening in-country diagnostic capacity for the testing of COVID-19 by leveraging and optimising three existing laboratories within the NCDC molecular laboratory network and assessing existing infectious disease treatment centres with a focus on identifying gaps and developing plans for case management. Interim protocols and guidelines for case management of COVID-19 were developed while the Nigeria Pandemic Influenza Preparedness and Response plan was reviewed for relevance to COVID-19 response. Infection prevention and control (IPC) and case management trainings were conducted for frontline health care workers in designated treatment centres.

Findings from a WHO risk assessment identified 13 countries, including Nigeria, as high-risk priority zones for proactive surveillance, detection and containment of the spread of COVID-19 [12]. Consequently, an in-country risk assessment was conducted to assess border screening at the country’s international airports, and surveillance efforts were enhanced at the four international airports across the country to include temperature checks for all passengers and screening questionnaires for passengers arriving from countries with community transmission of COVID-19. The NCDC also began to release updates on the outbreak and recommended preventive measures to the public. The first public health advisory was issued on January 22, 2020, with updated versions subsequently published on the NCDC website and disseminated using multiple streams including social media. In addition, to assess and test the functional capabilities of all response systems in terms of preparedness, the NCDC and its partners conducted a national multi-stakeholder simulation exercise on February 27 and 28, 2020.

### Nigeria’s outbreak response measures

Following the confirmation of the first COVID-19 case in Nigeria on February 27, 2020, the NCPG transitioned to a national multisectoral Emergency Operations Centre (EOC) at the NCDC. The EOC was activated at level three, the highest level of response in the country intended for public health emergencies requiring national coordination and use of all available resources for the response. The EOC comprises multiple pillars, including: coordination, surveillance and epidemiology, case management, laboratory, points of entry (PoE), IPC, risk communication, logistics, and research. POE and case management pillars are led by the Departments of Port Health Services and Hospital Services of the Federal Ministry of Health respectively. Sub-national EOCs were activated in both Lagos and Ogun states to coordinate the response in the first two affected states. National multidisciplinary rapid response teams (RRTs) were strategically deployed to the initial two states (Lagos and Ogun), plus FCT, and then to all states to strengthen coordination and response activities at the state and local government area (LGA) levels. The national RRTs, comprising NCDC staff and graduates/residents of the Nigeria Field Epidemiology and Laboratory Training Program (NFELTP), provided technical and logistical support at the state and sub-state levels.

At the national level, the Presidential Task Force (PTF) on COVID-19 was established by the President of Nigeria on March 9, 2020, with an overarching mandate to coordinate and oversee the country’s multi-sectoral and inter-governmental efforts both to contain the outbreak and to mitigate the impact of the COVID-19 pandemic in Nigeria. The National COVID-19 Multi-Sectoral Pandemic Response Plan was adopted by the PTF in March and serves as a blueprint for a whole-of-Government response.

The PTF provided high-level strategic leadership to the national response guided by scientific evidence. Daily PTF media briefings were held to enlighten Nigerians on evolving evidence, address trending issues and provide update on the government’s response. Technical evidence-based recommendations from the PTF informed the President of Nigeria’s policy decisions for the various phases of the outbreak. Overall, Nigeria’s response strategies were aimed at suppressing the transmission of COVID-19 by testing all suspect cases, isolating all confirmed cases, and tracing all contacts of confirmed cases, with the implementation of country-wide or regional non-pharmaceutical interventions as appropriate. The Nigerian response was characterised by robust collaborations with partners. Development and implementation of response strategies were facilitated by technical and material support from several local and international partners including the WHO, Africa CDC and philanthropic organisations. The need to generate relevant research evidence led to the formation of the Nigeria COVID-19 Research Consortium (NCRC) whose aim is to develop and implement a research agenda on COVID-19 with identified national priorities, in line with WHO’s global research roadmap. NCRC also serves as the coordinating body for COVID-19 research in Nigeria.

Given the novelty of the virus, the evolving nature of transmission in Nigeria from imported cases to clusters of cases to community transmission and level of response implemented, the NCDC EOC convened a mid-action review meeting on May 9, 2020, to strategically review the existing response approach, share lessons learnt, and identify key opportunities for improvement and further collaboration. The outcome and key recommendations of the meeting in line with the emerging data and global best practices have been used to improve response strategies, drive control and prevention measures against the disease, as well as focused interventions to strengthen the health system. The gaps identified include poor utilization of state level public health EOCs for coordinated responses, sub-optimal utilization of data to guide decision making, delayed turn-around-times of laboratory results, non-standardization of case management across treatment centers and poor adherence to IPC practices in health care facilities. Several intervention measures were instituted which include training and mentorship of State EOC teams on the incident management system as a tool for outbreak response coordination, development and operationalization of data management, analysis and use plans, deployment of the electronic surveillance system to laboratories to speed up the release of results, the establishment of a community of practice for COVID-19 case managers and deployment of online IPC training programme for health care workers.

## RESPONSE STRUCTURES

### Travels, lockdowns and COVID-19 spread

**Figure 2:**
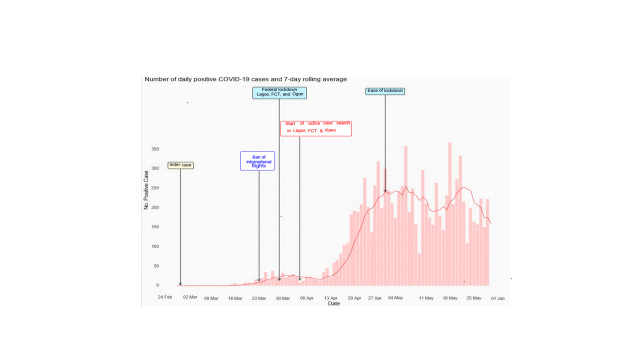
Epidemic curve of laboratory confirmed COVID-19 cases by date of reporting with mitigation strategies implemented in Nigeria, 2020.

As of March 22, 2020, the initial 30 confirmed cases COVID-19 in Nigeria were travellers from abroad or their immediate contacts. This informed the initial international travel ban for passengers coming from countries with ongoing high transmission (initially China, Italy and Germany; subsequently extended to eight high-burden countries) to minimize rising imported cases. Ultimately, land borders were closed, all international flights were banned, and mandatory institutional quarantine and testing for international returnees to Nigeria was instituted on March 23, 2020 to reduce further importation of the disease from high-risk countries (**Figure 2**).

On March 30, 2020, the President of Nigeria issued a series of stringent non-pharmaceutical interventions, including stay-at-home orders and cessation of non-essential movements and activities (collectively referred to as a “lockdown strategy”) in Lagos and Ogun States and FCT for an initial period of 14 days, extended for an additional 21 days in the same three states and adding Kano State. The states were selected based on a combination of the burden of disease and their risk: Lagos State was the initial epicentre of disease and had the highest number of cases; Ogun State borders Lagos State, was the source of the index case, and has a highly urban population with a high rate of travel into Lagos State; the FCT had the second-highest number of cases at that time. After the initial two-week lockdown period, incidence in Kano increased rapidly, prompting inclusion in the lockdown. The lockdown included closure of schools and workplaces, bans on religious and social gatherings, cancellation of public events, curfews, restrictions on movement, and cessation of interstate and international travel. Alongside the federal lockdown in Lagos, and Ogun States and the FCT, many states adopted measures as well, including school closure, movement restrictions, and curfews.

The lockdown strategy was a drastic and temporary measure implemented with two objectives: first, to slow the spread of the virus across the country, and second, to buy time for the health system to increase its preparedness. During the lockdown period, the NCDC worked with all states to enhance contact tracing activities and increase capacity for case detection and treatment. Treatment centres were expanded from an initial single centre in Lagos with 35 beds, as of February 29, 2020, to 38 centres with 1055 beds by April 14; by May 30, 2020 Nigeria had 121 treatment centres with 6550 beds. In the four-week period, the number of laboratories able to carry out COVID-19 testing increased from the initial three to 13 laboratories in 10 states as of April 15, to 28 in 18 states by the end of May. More than 13 000 health care workers were trained on IPC as well as on COVID-19 case management and personal protective equipment (PPE) and response commodities were deployed across the country to reinforce and better prepare the multi-sectoral response.

Despite bans on interstate travel, the virus had already spread geographically. Ten states reported their first COVID-19 cases during the first 14-day phase of the Federal lockdown, while an additional 13 states reported index cases in the second phase of the lockdown. Index cases in several states were traced to domestically exported cases from Lagos State and FCT. Nearly three-quarters (74%, n = 7532) of current cases have no known epidemiological link, suggesting substantial community transmission. Cumulatively, as of May 31, 2020, 337 of Nigeria’s 774 LGA have reported a confirmed case.

Although laboratory capacity was scaled up rapidly, testing numbers did not increase as planned. Testing capacity in terms of PCR machines as of mid-April was approximately 2500 tests per day and increased to more than 5000 per day as three Mega PCR Laboratories were activated for COVID-19 testing in Lagos and FCT by late May. However, inadequate testing reagents, due to delayed orders, and airport closures, hindered testing scale-up. Limited numbers of sample collection teams and testing centres, as well as hesitancy among some of the population to test, have also constrained increased testing. In the first two weeks of April, on average fewer than 300 tests per day were conducted; this increased to approximately 640 per day in the last two weeks of April, 1145 per day during the first two weeks of May, and 1410 per day during the last two weeks of May.

### Easing of lockdown

The drastic lockdown measures came with a significant economic and social cost. Crime and domestic violence reportedly increased during the period [13] and many people were unable to exercise their usual income-generating activities with effects most pronounced on vulnerable populations and those living in poverty [14]. Upon the completion of five weeks of a federally mandated lockdown, a gradual segmented easing of lockdown measures was initiated on May 4, 2020. This was a phased approach for an initial period of two weeks to create a balance between public health and economic consequences by progressively returning the population to normal activities. This easing of lockdown measures was supplemented by increased testing and contact tracing by rapid response teams, testing centres, and state public health department teams. On March 30, the eve of the lockdown, 71% of contacts of confirmed cases were followed up; by the end of May, this had increased to 91%. The nationwide mitigation measures implemented by the government of Nigeria in the first two weeks post-lockdown includes an 8:00 pm to 6:00 am curfew, mandatory use of face masks in public, a continued ban on interstate and international movement, prohibition of mass gatherings of more than 20 people, and mandatory testing and supervised isolation of at least 14 days for repatriated citizens.

## DISCUSSION

Globally, countries continue to employ diverse strategies to control the COVID-19 pandemic. These strategies are aimed at preventing, detecting, controlling, and mitigating the impact of the pandemic while taking into account the economic, social, cultural and religious factors unique to each country. Ultimately, the effectiveness of these strategies will be determined and sustained by country-level and community ownership [15].

Nigeria has, to date, the second-highest number of confirmed COVID-19 cases in Africa, and accounts for 7% of all confirmed cases on the continent. This may be an underestimate of the actual case load given the relatively low testing rate in Nigeria. As of May 31, Nigeria had conducted 63 882 COVID-19 tests, equivalent to 293 tests per million population; in comparison, Ghana had conducted 184 343 (5948 per million population) and South Africa had conducted 488 609 tests (8251 per million population) [16]. The relatively low CFR could be attributable to the robust case management measures coupled with a favourable demographic profile of the country, with 70% of the population below 25 years of age.

By the end of May 2020, all but one state, Cross River State, reported COVID-19 cases. Cases were concentrated in the two most densely populated states, Lagos and Kano, as well as the FCT. Many of the initial cases reported outside of these three states have been imported from Lagos State, the FCT, or Kano State. Meanwhile, the rising case numbers in conflict-affected areas such as Borno State pose additional concerns and require context-specific interventions to avert a looming health crisis, especially in camps for internally displaced persons [16,17].

Undetected imported cases may have played a key role in the beginning and accelerated the transition from clustered to community transmission in the Nigeria outbreak. This is because, as the outbreak began, prior to closure of international borders, arriving international passengers were allowed to self-quarantine (without testing) but encouraged to seek testing if symptoms developed. This may have led to multiple undetected imported cases. The rapid emergence of cases among individuals with no travel history or recognized epidemiological links to other cases despite contact tracing suggests multiple undetected cases may have been imported into the country or that potential contacts of the identified cases were missed. This guided subsequent approach such that following border closure, arriving passengers (eg, on repatriation flights) had mandatory supervised quarantine for 14 days.

Nigeria’s states were facing critical challenges as the number of cases continues to increase across the country. Foremost among these is the limited number of COVID-19 treatment centres. Though greatly expanded since the outbreak’s start, the limited number of beds, health workers, and critical care equipment such as oxygen and ventilators may quickly lead to an overwhelmed health system unable to minimize COVID-19 mortality and contain the spread of the disease. The NCDC continues to work closely with all state governments and partners to ensure adequate infrastructure is in place for timely case detection, management of cases, and to build capacity of the public health and clinical workforce.

Although national testing capacity has significantly increased with private sector engagement, testing coverage and pace is still relatively low. By the end of May 2020, 293 tests had been conducted per million population, about 5% of what Ghana had done, with an average positivity rate of 15.9% overall. Nigeria needs to rapidly scale up testing to be able to quickly identify existing cases, isolate them, and control the pandemic. Securing sufficient testing supplies and personal protective equipment for health care personnel are immediate challenges of the response.

Lock down containment measures were aimed at slowing the spread of the outbreak to new states, delaying the progression to community transmission, and increasing health system capacity at the initial phase of the outbreak. Though the lockdown slowed down COVID-19 transmission, it had undesired collateral effects on social protection, security, and daily subsistence for many. It is safe to assume that these negative consequences of the pandemic disproportionately affected women, people living in poverty, petty traders and those dependent on income from small and medium enterprises. The adverse effects of the lockdown exacerbated already difficult situations for many, rendering prolonged enforcement of preventive interventions such as lockdown and physical distancing unsustainable.

A systematic approach was adopted to balance economic reopening with public health concerns, demonstrated in the phased easing of lockdown alongside a whole-of-society outbreak control strategy. This approach mobilises all sectors and communities to take ownership of and responsibility for response efforts towards control of the war against COVID-19 in Nigeria. The easing of lockdown measures came at a time when confirmed cases were on the uptick; however, the immense socioeconomic impact witnessed during the lockdown period necessitated a more balanced approach to public health interventions [17]. Furthermore, the easing of the federal lockdown allowed for a state-owned approach consistent with Nigeria’s governance structure and health system.

## CONCLUSION

Nigeria mounted a swift and aggressive response to COVID-19, leveraging on its existing epidemic preparedness and learning from other parts of the globe where transmission began earlier. The country’s initial response included early activation of the national EOC at the NCDC, establishment of the multi-sectoral COVID-19 PTF, and decisive actions to stop international travel and impose a time-limited lockdown in highly affected areas. By rapidly implementing this set of interventions, Nigeria likely slowed down the rate of virus transmission and bought extra time to implement a robust case detection, testing, and treatment centre capacity. However, these efforts, especially testing, needs more private sector involvement to significantly ramp up COVID-19 diagnostic centres across the country. Sensitising and mobilising citizens to take responsibility by strict implementation of preventive non-pharmaceutical measures is key to flattening the curve. A rapid, holistic, cohesive, whole-of-government approach that encompasses civil society and local-communities in the response will be absolutely critical to combating the COVID-19 pandemic in Nigeria [18] and rebuilding stronger health systems towards adjusting to a “new normal”.
